# An EP2 Agonist Facilitates NMDA-Induced Outward Currents and Inhibits Dendritic Beading through Activation of BK Channels in Mouse Cortical Neurons

**DOI:** 10.1155/2016/5079597

**Published:** 2016-05-19

**Authors:** Yoshinori Hayashi, Saori Morinaga, Xia Liu, Jing Zhang, Zhou Wu, Takeshi Yokoyama, Hiroshi Nakanishi

**Affiliations:** ^1^Department of Aging Science and Pharmacology, Faculty of Dental Sciences, Kyushu University, Fukuoka 812-8582, Japan; ^2^Department of Dental Anesthesiology, Faculty of Dental Sciences, Kyushu University, Fukuoka 812-8582, Japan; ^3^Institute for Metabolic and Neuropsychiatric Disorders, Binzhou Medical University, Binzhou 256603, China

## Abstract

Prostaglandin E_2_ (PGE_2_), a major metabolite of arachidonic acid produced by cyclooxygenase pathways, exerts its bioactive responses by activating four E-prostanoid receptor subtypes, EP1, EP2, EP3, and EP4. PGE_2_ enables modulating* N*-methyl-D-aspartate (NMDA) receptor-mediated responses. However, the effect of E-prostanoid receptor agonists on large-conductance Ca^2+^-activated K^+^ (BK) channels, which are functionally coupled with NMDA receptors, remains unclear. Here, we showed that EP2 receptor-mediated signaling pathways increased NMDA-induced outward currents (*I*_NMDA-OUT_), which are associated with the BK channel activation. Patch-clamp recordings from the acutely dissociated mouse cortical neurons revealed that an EP2 receptor agonist activated *I*_NMDA-OUT_, whereas an EP3 receptor agonist reduced it. Agonists of EP1 or EP4 receptors showed no significant effects on *I*_NMDA-OUT_. A direct perfusion of 3,5′-cyclic adenosine monophosphate (cAMP) through the patch pipette facilitated *I*_NMDA-OUT_, which was abolished by the presence of protein kinase A (PKA) inhibitor. Furthermore, facilitation of *I*_NMDA-OUT_ caused by an EP2 receptor agonist was significantly suppressed by PKA inhibitor. Finally, the activation of BK channels through EP2 receptors facilitated the recovery phase of NMDA-induced dendritic beading in the primary cultured cortical neurons. These results suggest that a direct activation of BK channels by EP2 receptor-mediated signaling pathways plays neuroprotective roles in cortical neurons.

## 1. Backgrounds

We have previously reported that interleukin-1*β* (IL-1*β*) increases neuronal excitability via the inhibition of large-conductance Ca^2+^-induced K^+^ channels (BK) activated by Ca^2+^ influx through both NMDA receptors (NMDARs) and voltage-dependent Ca^2+^ channels (VDCC) in the dissociated hippocampal neurons [1, 2]. The inhibitory effect of IL-1*β* on the amplitude of NMDA-induced outward currents (*I*_NMDA-OUT_), which is mediated by BK channel activation, is largely mediated by phosphorylation of p38 mitogen-activated protein kinase (MAPK) [2]. It is considered that active p38 MAPK directly phosphorylates BK channels to decrease the amplitude of *I*_NMDA-OUT_. On the other hand, it has been reported that prostaglandin E_2_ (PGE_2_) potentiates the amplitude of *I*_NMDA-OUT_ in the dissociated preoptic neurons [3]. However, the precise intracellular mechanism underlying the effects of PGE_2_ on BK channels activated by Ca^2+^ influx through NMDARs still remains to be determined.

PGE_2_, a major metabolite of arachidonic acid produced by cyclooxygenase pathways, can bind to four E-prostanoid receptor subtypes, EP1, EP2, EP3, and EP4. The EP1 receptors couple to Gq and unidentified G protein. The EP2 and EP4 receptors couple positively to Gs to increase cAMP, whereas the EP3 receptors couple negatively to cAMP via Gi [4]. PGE_2_ has a potential therapeutic role for the neurotoxicity through EP2 receptors. In addition, the selective EP2 receptor activation leads to a protection of neurons against NMDAR-mediated excitotoxicity and oxygen-glucose deprivation-induced anoxia in the hippocampal neurons [5–7], whereas the genetic deletion of EP2 receptors exacerbates the brain damage after infarction [8]. At present, it is considered that EP2 receptors induce neuroprotection through the activation of cAMP-protein kinase A (PKA) pathway and the subsequent activation of cAMP-responsive element binding protein (CREB), which facilitates the neuronal survival [9]. In fact, PKA inhibitors abrogate the EP2 receptor-mediated neuroprotection [5].

In the present study, we showed that PGE_2_ enhanced *I*_NMDA-OUT_ through the activation of EP2 receptors in mouse cortical neurons. The activation of cAMP/PKA signaling pathway was involved in the EP2 receptor agonist-induced potentiation of *I*_NMDA-OUT_. Furthermore, an EP2 receptor agonist facilitated the recovery from NMDA-induced dendritic beading.

## 2. Materials and Methods

### 2.1. Animals

The experimental protocol was approved by the Animal Research Committee of Kyushu University. All efforts were made to minimize animal suffering and to reduce the number of animals used. Embryonic day 19 mouse pups and 3-week-old male C57BL/6 mice were used for electrophysiology and immunohistochemistry. The mice were maintained on a 12 hr light/dark cycle (light on at 8:00 AM) under conditions of 22–25°C ambient temperature with food and water* ad libitum*.

### 2.2. Compounds

KT5720 (a specific PKA inhibitor), cAMP, NMDA, and paxilline (a specific BK channel blocker) were purchased from Sigma. Butaprost (an EP2 receptor agonist) and TG6-10-1 (an EP2 receptor antagonist) were purchased from Cayman and Millipore, respectively. The concentrations of EP2 agonists/antagonists were determined according to the paper as previously described [5, 10]. Synthesized selective agonists of EP1 receptor (ONO-DI-004), EP2 receptor (ONO-AE1-259), EP3 receptor (ONO-AE-248), and EP4 receptor (ONO-AE1-329) were kindly provided from Ono Pharmaceuticals.

### 2.3. Cortical Slice Preparation and Mechanical Dissociation of Cortical Neurons

The C57BL/6 mice (3 weeks old) were anesthetized with pentobarbital sodium (200 mg/kg i.p.) and then decapitated. The cortical slice preparations with a thickness of 375 *μ*m were made using a microslicer (VT1000S; Leica, Nussloch, Germany). The slices were kept in the incubation medium saturated with 95% O_2_-5% CO_2_ at room temperature (21–24°C) for ≥1 hr before mechanical dissociation. The incubation medium contained (mM) 124 NaCl, 3 KCl, 1.5 KH_2_PO_4_, 24 NaHCO_3_, 2.4 CaCl_2_, 1 MgCl_2_, and 10 glucose. Mechanical dissociation was accomplished using a custom-built vibration device and a fire-polished glass pipette as previously described [11].

### 2.4. Patch-Clamp Recording

Voltage-clamp recordings from mechanically dissociated cortical neurons were carried out using perforated whole-cell patch-clamp technique as previously described [1]. Amphotericin B (260 *μ*g/mL) was added to pipette solution (the pipette solution contained (mM) 70 K-methanesulfonate, 10 KCl, and 10 HEPES). The external solution contained (in mM) 150 NaCl, 5 KCl, 2 CaCl_2_, 10 HEPES, and 10 glucose (pH 7.4). For the whole-cell recordings, cAMP or KT 5720 was added to pipette solution. NMDA was applied for 20 sec at an interval of 5 min [11]. Agonists of EP1–EP4 receptors were simultaneously applied with NMDA. The holding potential was maintained at −40 mV throughout the voltage-clamp experiments.

### 2.5. Primary Cultured Cortical Neurons

Cortices were obtained from embryonic day 19 (E19) mouse pups and dissociated with 90 U papain (Worthington Biochemical Corp., Lakewood, NJ, USA) in Hank's balanced salts solution (HBSS) (Gibco, Grand Island, NY USA) containing 10 mM N-[2-hydroxyethyl]piperazine-N′-[2-ethanesulfonic acid] (HEPES) (pH 7.5). Cortices were seeded onto polyethyleneimine (PEI) coated 13*ϕ* cover glass (Matsunami) with attachment medium. Medium was replaced into maintenance medium on the following day. Attachment medium was minimum essential medium (MEM) (Gibco) containing 10% horse serum, 450 mg/mL glucose, 1% penicillin/streptomycin, and 1% sodium pyruvate. Maintenance medium was neurobasal medium containing 2% B-27 (Gibco), 1% glutamine, and 1% penicillin/streptomycin. Cortical neurons were used for the experiment on 10 days* in vitro* (DIV).

### 2.6. Immunohistochemistry

NMDA (30 *μ*M) was diluted with Locke's solution and applied to cortical neurons on 10 DIV at 37°C for 10 minutes. Locke's solution contained (mM) 154 NaCl, 5.6 KCl, 2.3 CaCl_2_, 1 MgCl_2_, 3.6 NaHCO_3_, 5 HEPES, and 10 glucose (pH 7.2). NMDA exposure was terminated by fixation with 4% paraformaldehyde at 4°C for 1 hr. Cortical neurons on the 13*ϕ* cover glass were permeabilized with 0.4% Triton X-100 for 1 hr on 24-well plate (Corning). Cortical neurons were incubated antimicrotubule associated protein 2 (MAP-2, 1 : 800, Millipore) overnight at 4°C and then incubated with secondary antibody conjugated with Alexa 488 (1 : 400, Jackson Immunoresearch) at 4°C for 2 hr. Cortical neurons were mounted in the antifading medium Vectashield (Vector Laboratories). Images were captured with the C2si Confocal Laser Microscope (CLMS, Nikon Corporation, Tokyo, Japan) using ×20 (NA: 0.75) dry lens and ×60 (NA: 1.4) oil immersion lens.

### 2.7. Analyses of Dendrite Morphology in Cortical Neurons

Bead-forming neuron was defined as the neuron that has at least one beading structure on the dendrite. The number of bead-forming neurons was counted. Bead formation was displayed as a percentage of bead-forming neurons/total neurons in randomly captured images from 20 fields.

### 2.8. Data Analysis

The data are represented as the mean ± SEM. Statistical analyses of the results were performed with one-way analysis of variance (ANOVA) with* post hoc* Dunnett's or Tukey's test and two-way ANOVA with Bonferroni test or unpaired* t*-test using the GraphPad Prism software package. The data met the assumptions of equal variances. Differences were considered significant with *P* values less than 0.05.

## 3. Results

### 3.1. Effects of E-Prostanoid Receptor Agonists on *I*_NMDA-OUT_

**Figure 1:**
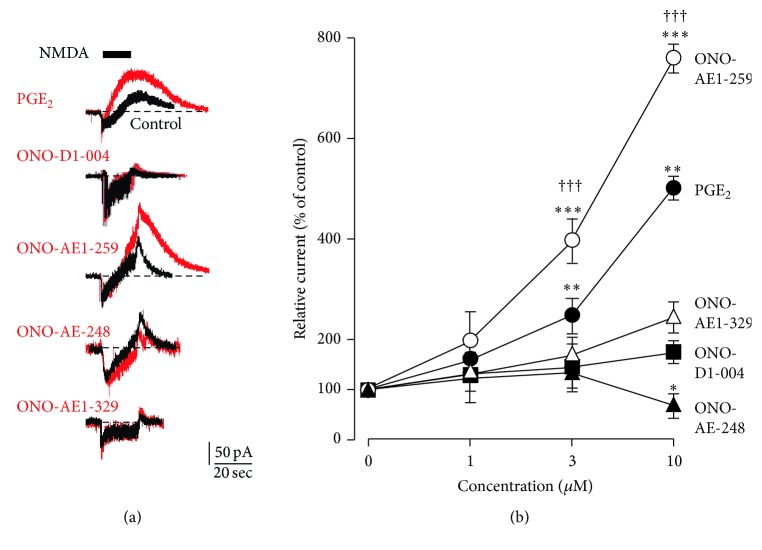
Potentiation of NMDA-induced outward currents through the activation of EP2 receptors in the acutely dissociated mouse cortical neurons. (a) Superimposed traces show NMDA-induced currents in the absence (black traces) or the presence (red traces) of drugs. PGE_2_ (10 *μ*M), ONO-DI-004 (an EP1 receptor agonist, 10 *μ*M), ONO-AE1-259 (an EP2 receptor agonist, 10 *μ*M), ONO-AE-248 (an EP3 receptor agonist, 10 *μ*M), and ONO-AE1-329 (an EP4 receptor agonist, 10 *μ*M) were simultaneously applied with NMDA (30 *μ*M). A black line indicates the drug application period. Broken lines indicate basal currents. Calibration bars are 50 pA and 20 sec. (b) Dose response of EP receptor agonists on NMDA-induced outward currents. The symbols and bars represent the mean ± SEM (*n* = 5–7 cells in each responses). Asterisks indicate a significant difference from 0 *μ*M (^*∗*^ *P* < 0.05, ^*∗∗*^ *P* < 0.01, and ^*∗∗∗*^ *P* < 0.001, one-way ANOVA with* post hoc* Dunnett's test). Swords indicate a significant difference between PGE_2_ and ONO-AE1-259 (^†††^ *P* < 0.001, *F*_1, 40_ = 47.28, two-way ANOVA Bonferroni test).

The direct effects of four E-prostanoid receptor agonists on *I*_NMDA-OUT_ were analyzed. Amphotericin B-perforate patch-clamp recordings from acutely dissociated mouse cortical neurons were conducted to minimize the intracellular dialysis, because the activation of BK channels by Ca^2+^ influx through NMDARs is the underlying mechanism of *I*_NMDA-OUT_ [12]. PGE_2_ and selective E-prostanoid receptor agonists were applied 5 min before and during the application of NMDA. NMDA (30 *μ*M) application for 20 sec at the holding potential of −40 mV elicited inward currents (*I*_NMDA-IN_) and subsequent outward currents (*I*_NMDA-OUT_) shown in Figure 1(a), similar to our previous observations [1]. PGE_2_ (1–10 *μ*M) significantly increased the mean amplitude of *I*_NMDA-OUT_ in a dose dependent manner. ONO-AE1-259 (1–10 *μ*M), an EP2 receptor agonist, was found to mimic the effect of PGE_2_ (Figures 1(a) and 1(b)). It was also noted that the increasing effect of an EP2 receptor agonist on the mean amplitude of *I*_NMDA-OUT_ was significantly stronger than that of PGE_2_ (*P* < 0.001, *F*_1, 40_ = 47.28, two-way ANOVA; Figure 1(b)). In contrast, ONO-AE-248 (10 *μ*M), an EP3 receptor agonist, significantly reduced the amplitude of *I*_NMDA-OUT_ (*P* < 0.05, one-way ANOVA with* post hoc* Dunnett's test; Figures 1(a) and 1(b)). On the other hand, ONO-DI-004 and ONO-AE1-329 (1–10 *μ*M), EP1 and EP4 receptors agonists, respectively, showed no significant effects on *I*_NMDA-OUT_ (Figures 1(a) and 1(b)).

### 3.2. Involvement of cAMP/PKA Signaling Pathway in EP2 Agonist-Induced Potentiation of *I*_NMDA-OUT_

**Figure 2:**
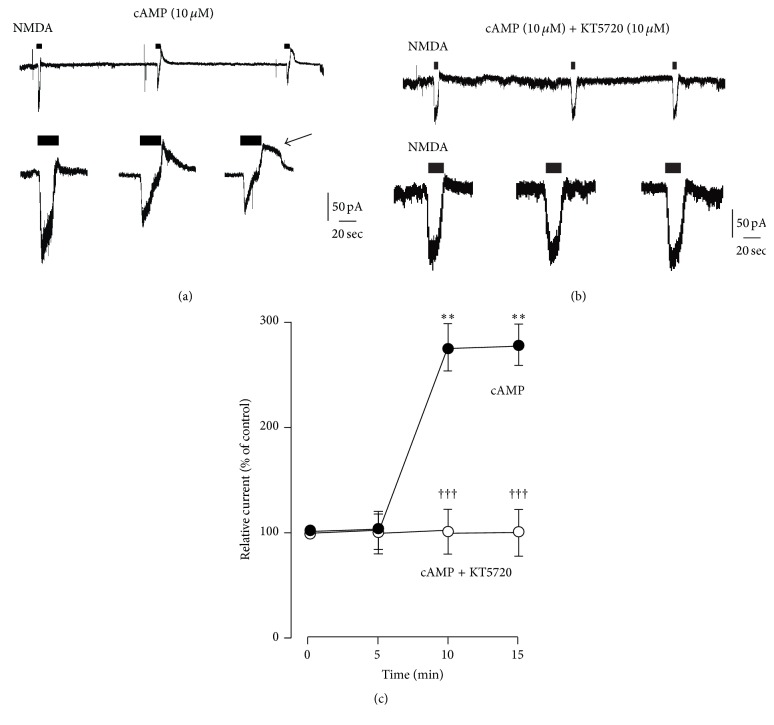
Cyclic AMP/PKA pathway facilitates NMDA-induced outward current in the acutely dissociated mouse cortical neurons. (a, b) Intracellular perfusion of cAMP (10 *μ*M) facilitated NMDA-induced outward currents (a), whereas KT5720 (10 *μ*M), a specific inhibitor of PKA, abolished them (b). Upper trace is continuous recording of NMDA-induced currents. Lower traces are enlarged traces of upper trace. An arrow indicates the prolonged NMDA-induced outward current. Calibration bars are 50 pA and 20 sec applicable to lower traces. (c) Time course of the potentiation of NMDA-induced outward currents by intracellular perfusion of cAMP. The circles and bars represent the mean ± SEM (*n* = 4 cells). Asterisks indicate a significant difference from the none-treated control (^*∗∗*^ *P* < 0.01, one-way ANOVA with* post hoc* Dunnett's test). Swords indicate a significant difference between cAMP and cAMP+KT5720 (^†††^ *P* < 0.01, *F*_1, 24_ = 39.32, two-way ANOVA Bonferroni test).

**Figure 3:**
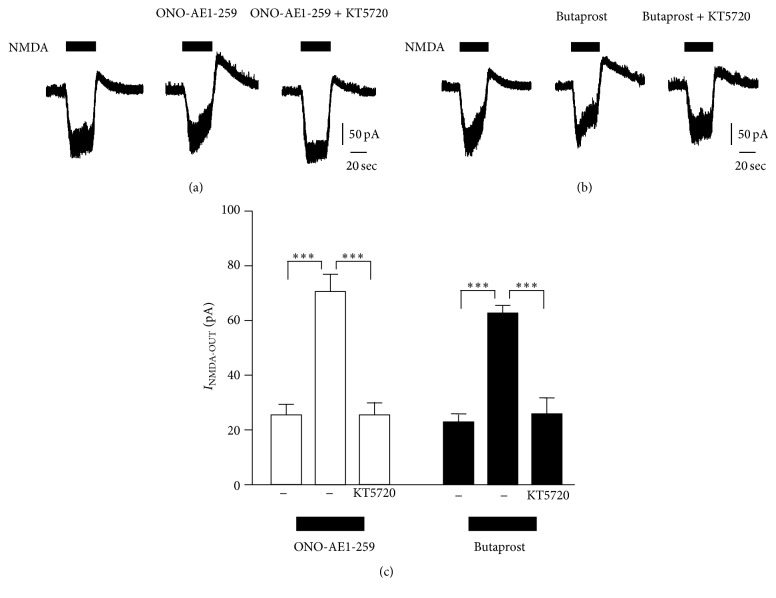
Inhibitory effects of PKA inhibitor on EP2 receptor agonists-induced potentiation of *I*_NMDA-OUT_. (a, b) KT5720 (10 *μ*M) inhibited ONO-AE1-259 (10 *μ*M) or butaprost- (10 *μ*M) induced potentiation of *I*_NMDA-OUT_. (c) Averaged amplitude of NMDA-induced outward currents. The columns and bars represent the mean ± SEM (*n* = 3 cells). Asterisks indicate a significant difference between the values (^*∗∗∗*^ *P* < 0.001, unpaired* t*-test). Calibration bars are 50 pA and 20 sec.

The activation of EP2 receptors stimulates cAMP synthesis, which in turn activates PKA [4]. We next ask whether EP2 receptor signaling pathways really can potentiate the amplitude of *I*_NMDA-OUT_. To address the requirement of increased intracellular cAMP levels on the amplitude of *I*_NMDA-OUT_, we performed intracellular application of cAMP through the patch pipette. Even in the whole-cell configuration, *I*_NMDA-OUT_ at holding potential of −40 mV was observed in the acutely dissociated mouse cortical neurons, similar to previous observations [2]. The mean amplitude of *I*_NMDA-OUT_ was significantly increased 10 minutes after intracellular perfusion of cAMP (10 *μ*M) (Figures 2(a) and 2(c)). At the same time, the duration of *I*_NMDA-OUT_ was markedly prolonged (Figure 2(a), an arrow). cAMP-induced increase in the amplitude and duration of *I*_NMDA-OUT_ was completely inhibited by KT5720, a specific inhibitor of PKA (Figures 2(b) and 2(c)). We further analyzed the involvement of PKA signaling on EP2 receptor agonists-induced potentiation of *I*_NMDA-OUT_. To address this, we utilized distinct types of EP2 receptor agonist, ONO-AE1-259 and butaprost. An increase in the amplitude of *I*_NMDA-OUT_ by EP2 receptor agonists was significantly inhibited by perfusion of KT5720 through the patch pipette (Figure 3). These results suggest that EP2 receptor signaling pathway facilitates the amplitude of *I*_NMDA-OUT_ through PKA signaling pathway.

### 3.3. Facilitation of the Recovery Phase of NMDA-Induced Dendritic Beading by the Activation of EP2 Receptors

**Figure 4:**
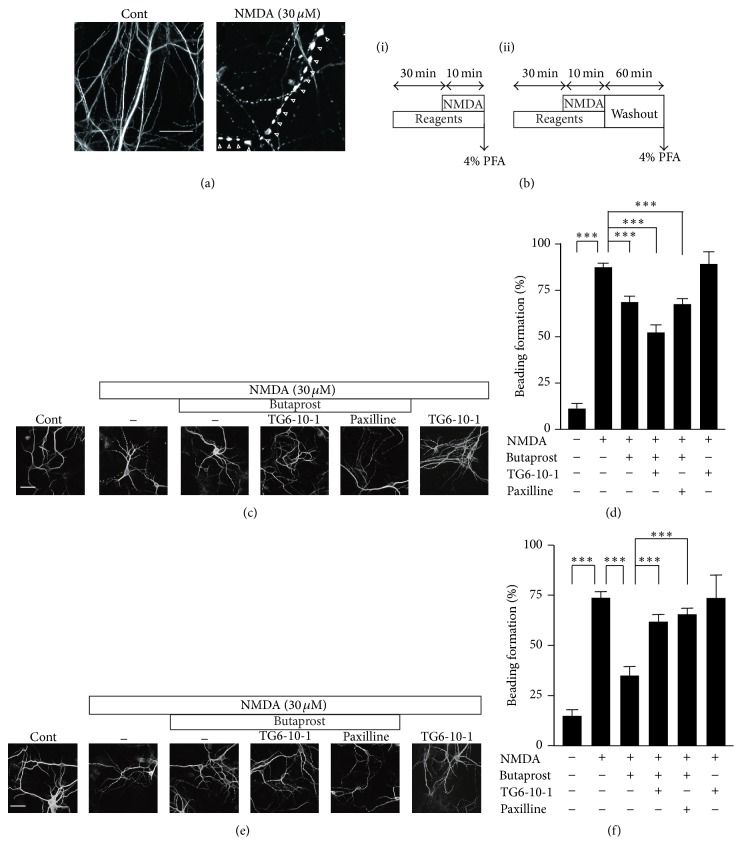
The facilitatory effects of butaprost, an EP2 receptor agonist, on the recovery phase of NMDA-induced dendritic beading through the activation of BK channels in the primary cultured mouse cortical neurons. (a) Immunofluorescent CLMS images for MAP2 in the primary cultured cortical neurons of pretreatment (left panel) or posttreatment (right panel) of NMDA (30 *μ*M) for 10 min. Arrowheads indicate beads-structure in the dendrite. Scale bar = 20 *μ*m. (b) Cortical neurons were fixed 10 min after the stimulation with NMDA (i) or 60 min after the elimination of NMDA (ii). Experimental schedules of (i) and (ii) were corresponding to (c, d) and (e, f), respectively. Data are represented as the mean ± SEM (*n* = 180–235 cells). (c, e) Immunofluorescent CLMS images for MAP2 in the primary cultured cortical neurons at 10 min after stimulation of NMDA (c) or 60 min after the elimination of NMDA (e). Butaprost (1 *μ*M), TG6-10-1 (10 *μ*M), an EP2 receptor antagonist, and paxilline (2 *μ*M) were treated 30 min before the application of NMDA. (d, f) The formation of dendritic beading of 10 min after the stimulation with NMDA (d) or 60 min after the elimination of NMDA (f). The columns and bars represent the mean ± SEM (*n* = 142–173 cells). Asterisks indicate a significant difference between the values (^*∗∗∗*^ *P* < 0.001, one-way ANOVA* post hoc* Tukey' test).

We finally examined effects of an EP2 receptor agonist on NMDA-induced dendritic beading in the primary cultured mouse cortical neurons, because dendritic beading is an early morphological hallmark of excitotoxic neuronal injury. Dendritic beading leads to disappearance of dendritic spines and neurite breakage, culminating in neuronal death [7, 13, 14]. Immunofluorescent CLMS images for MAP2 in the primary cultured cortical neurons showed that the application of NMDA (30 *μ*M) for 10 min induced the segmental focal swelling (dendritic beading) and loss of spines in the dendritic shafts (Figure 4(a)). Butaprost, an EP2 receptor agonist, partially inhibited NMDA-induced dendritic beading (Figures 4(c) and 4(d)). It is known that NMDA-induced dendrite beads disappeared completely 120 min after the elimination of NMDA [13]. Thus, it is possible that EP2 receptor agonist-mediated neuroprotection is mainly due to the facilitation of recovery phase of NMDA-induced dendritic beading. Under the none-treated control condition, NMDA-induced dendritic beading remained even at 60 min after washout of NMDA (Figures 4(e) and 4(f)). When butaprost was applied with NMDA, dendritic beading was significantly recovered at 60 min after washout of NMDA (Figures 4(e) and 4(f)). TG6-10-1, an EP2 receptor antagonist, significantly inhibited the butaprost-induced facilitation of recovery. We further analyzed the relationship between EP2 receptors and BK channels on neuroprotection. Paxilline, a specific BK channel blocker, also significantly abolished butaprost-induced facilitation of recovery (Figures 4(e) and 4(f)). Therefore, it is conceivable that the activation of BK channels is responsible for an EP2 agonist-induced facilitation of recovery from NMDA-induced dendritic beading.

## 4. Discussion

In the present study, an EP2 receptor agonist facilitates NMDA-induced outward currents through the activation of BK channels. cAMP/PKA signaling pathway potentiates the amplitude of *I*_NMDA-OUT_. Furthermore, EP2 receptor agonist-mediated activation of BK channels promotes the recovery phase of NMDA-induced dendritic beading. Therefore, the activation of BK channels may be responsible for the neuroprotective role of EP2 receptors against excitotoxicity in cortical neurons.

We observed distinct potency of PEG_2_ and ONO-AE-259-01 on *I*_NMDA-OUT_. This might be due to the distinct binding affinity for EP2 receptor. Ki value of PGE_2_ exhibited 12 nM [15], whereas that of ONO-AE-259-01 was 3 nM [16]. From the above observations, a potent effect of ONO-AE-259-01 compared with PGE_2_ on *I*_NMDA-OUT_ was reasonable. The affinity of ONO-AE-259-01 for other types of prostanoid receptors is comparatively low (Ki = 140 nM for prostaglandin D_2_ receptor, Ki > 3.3 × 10^3^ nM for EP1 receptor, EP3 receptor, EP4 receptor, prostaglandin F receptor, prostacyclin receptor, and thromboxane receptor) [16]. Based on the binding affinity of ONO-AE-259-01, the facilitatory effect of ONO-AE-259-01 on *I*_NMDA-OUT_ is mediated by EP2 receptors.

EP2 and EP4 receptors are involved in the intracellular increase in cAMP and subsequent activation of PKA [4]. One of the important molecular targets of PKA is the transcription factor CREB that promotes neuronal survival [9]. In fact, the protective effects of EP2 and EP4 receptor agonists on neuronal damage associated with ischemia, Alzheimer's disease, and epilepsy were reported [5, 6, 17, 18]. Despite similar downstream signaling pathways of EP2 and EP4 receptors, EP4 receptors agonist had no significant effect on the amplitude of *I*_NMDA-OUT_, suggesting a relatively low expression level of EP4 receptors in cortical neurons. These data are consistent with the region specific expression patterns of EP1 and EP4 receptors [14, 19, 20]. By contrast, EP3 receptors are broadly expressed in the cerebral cortex, hippocampus, and midbrain [21]. In the present study, we showed opposite effects of EP2 and EP3 receptor agonists on the amplitude of *I*_NMDA-OUT_ and BK currents in the cortical neurons. Distinct responsiveness of EP2 and EP3 receptor agonists to *I*_NMDA-OUT_ may account for the intracellular concentration of cAMP. Indeed, an EP3 receptor agonist conversely aggravates neuronal damage [22]. Thus, a local distribution pattern of EP receptor subtypes is critical for the functional outcomes of PGE_2_ on neurons.

Elevated levels of [Ca^2+^]_i_ and the membrane depolarization [23], which serve as a feedback regulators in neurons, are known to gate the BK channels. Sensitivity of BK channels to the voltage and [Ca^2+^]_i_ are modulated by protein phosphorylation of serine/threonine or tyrosine residues within the channels. Generally, PKA and PKG-mediated phosphorylation lead to the activation of BK channels, whereas PKC leads to the inhibition of BK channels [24]. Inside-out mode of single channel analyses has revealed that PKA makes transition of BK channels from close-state to open-state [25]. Thus, the amplitude of *I*_NMDA-OUT_ might be facilitated by the stimulation of EP2 receptors through the activation of PKA. The source of Ca^2+^ is considered as the endoplasmic reticulum (ER) or influx of extracellular calcium. Kaufmann et al. proposed that distinct route of Ca^2+^ activates BK channels [26] whose expression patterns are different from soma (clustered type) and extrasynaptic site (scattered type) [27]. The Ca^2+^ source of scattered type of BK channels might be the ER independent, whereas that of clustered type is internal stores [26]. Intriguingly, thapsigargin and ryanodine had no effect on the glutamate-evoked outward current which are mediated by BK channels [12]. From these observations, *I*_NMDA-OUT_ caused by NMDA-application might be emerging at extrasynaptic site. This is accountable for the mechanism for the beading formation as discussed below. Beading structure was formed on the dendrite, progressing toward to soma from the tip of dendrite [28], resulting in the neuronal damage. The facilitation of recovery phase of dendritic beading by an EP2 receptor agonist was mediated by the BK channels. Therefore, extrasynaptically located BK channels might respond to an EP2 receptor agonist. Among the NMDA receptors, NR2B containing NMDA receptors are involved in the excitotoxicity [29] and located in extrasynaptic site [30]. Given the Ca^2+^ source and NMDA receptor's properties, scattered type of BK channels might couple to NR2B. Thus, one of potential molecular targets of EP2 receptor agonist is scattered type of BK channels.

EP2 receptors are widely expressed in both neurons and glia [5], whereas they exhibit opposite effects in different cells. Administration of an EP2 receptor agonist immediately after status epileptics showed neuroprotective effects [31]. In contrast, conditional deletion of EP2 receptors in microglia attenuated inflammation in animal models of Parkinson's disease or Alzheimer's disease [32, 33]. These observations indicate that EP2 receptors in microglia serve as proinflammatory signaling in the chronic neuroinflammation. Zhang et al. reported that the expression level of* EP2* mRNA was increased in microglia after cellular activation [34]. In addition, autocrine/paracrine release of PGE_2_ further activates microglia to aggravate neuroinflammation [32, 33]. Likewise, IL-1*β* derived from microglia also has an essential role in neuroinflammation [35–37]. Microglia specific gene-ablation of EP2 receptors results in the reduction of IL-1*β* in the hippocampus during neuroinflammatory situation [32, 33]. Secreted IL-1*β* from microglia eventually causes a loss of feedback inhibition in neurons through the attenuation of BK channel activities [1, 2]. In this way, PGE_2_ and IL-1*β* synergistically worsen the pathology in chronic inflammatory situation. Taken together, the role of EP2 receptors on neuroprotection and neurodegeneration is determined by the period after the brain injury.

## 5. Conclusions

PGE_2_ enhanced *I*_NMDA-OUT_ through the activation of EP2 receptors in mouse cortical neurons. The activation of cAMP/PKA pathway is involved in the EP2 receptor agonist-induced potentiation of *I*_NMDA-OUT_. Furthermore, an EP2 receptor agonist facilitated the recovery from NMDA-induced dendritic beading. These results suggest a novel neuroprotective strategy using EP2 agonists against the acute excitotoxic damage.
